# Functional Outcomes and Quality of Life in Patients over 50 Years Old Treated with Quadriceps Tendon Repair: A Retrospective Study

**DOI:** 10.3390/jfmk11040381

**Published:** 2026-09-24

**Authors:** Salvatore Risitano, Alessia Fierro, Giorgio Borella, Antonio Milano, Stefano Artiaco, Marcello Capella, Pier Francesco Indelli, Alessandro Massè

**Affiliations:** 1 Department of Orthopaedic Surgery and Traumatology, Città della Salute e della Scienza, 10126 Turin, Italy; 2 Institute of Biomechanics, Paracelsus Medical University (PMU), 5020 Salzburg, Austria; 3 Department of Orthopaedics, Traumatology and Rehabilitation, University of Turin, 10126 Turin, Italy; 4 Centro Interdipartimentale di Scienze Mediche (CISMed)—Orthopaedics, Traumatology, University of Trento, 38122 Trento, Italy

**Keywords:** quadriceps tendon rupture, tendon repair, quadriceps tendinopathy, PROMs, IKDC, Lysholm score, SF-36

## Abstract

**Objective:** Quadriceps tendon rupture (QTR) is a rare but highly debilitating event, leading to a marked loss of knee extension and a significant reduction in joint function. While early surgical repair represents the treatment of choice, medium-term functional outcomes and the impact on quality of life are not yet fully defined. The aim of this study is to evaluate the clinical–functional outcomes and quality of life at a minimum of six months post-surgery in patients over 50 years of age who underwent quadriceps tendon repair. **Methods:** A retrospective study was conducted on patients of both sexes, aged between 50 and 80 years, who underwent surgical treatment for complete QTR at the CTO Hospital in Turin between 2018 and 2024. Demographic data and details regarding the type of surgical procedure were collected. All patients were re-evaluated at a minimum of 6 months post-surgery from a clinical perspective and using three validated questionnaires: the International Knee Documentation Committee (IKDC) Subjective Knee Evaluation Form, the Lysholm score, and the Short Form-36 (SF-36), to analyze joint function and quality of life, respectively. Additionally, the range of motion (ROM) of the operated knee, any extension deficit, and postoperative complications were assessed. **Results:** Of 61 patients who met the eligibility criteria, 29 (47.5%) completed all required questionnaires and were included in the final analysis (mean age 62 years; 27 males, 2 females). The mean BMI recorded at the time of injury was 28.6. The mean follow-up was 9.17 months. Two patients were treated with suture anchor repair, while 27 patients underwent reinsertion using transosseous tunnels. The mean post-operative flexion ROM was 102.4°. No tendon re-ruptures occurred. Two patients presented a residual extension lag. The mean IKDC score was 63.2 points. The SF-36 questionnaire revealed scores indicative of a good level of general well-being and a low psychological impact. The Lysholm questionnaire yielded a mean score of 83.15, indicating a good functional recovery of the operated limb. **Conclusions:** The obtained results suggest that quadriceps tendon repair in patients over 50 can achieve good functional outcomes and a low re-rupture rate, with a substantially preserved medium-term quality of life; however, given the retrospective, uncontrolled design and the exclusion of more than half of the eligible cohort, these findings should be interpreted with caution and confirmed by larger, prospective studies.

## 1. Introduction

Quadriceps tendon rupture (QTR) is a rare pathology with an estimated incidence of 1.37 cases per 100,000 inhabitants per year. It predominantly affects males over 40 years of age and represents one of the leading causes of knee extensor mechanism disruption [1]. The loss of tendon continuity leads to marked functional limitation, resulting in a significant reduction in ambulation capacity and activities of daily living [2].

Quadriceps tendon rupture is frequently associated with systemic pathologies that promote tendon degeneration, including diabetes, chronic renal failure, hyperparathyroidism, obesity, gout, and the chronic use of corticosteroids or fluoroquinolones [2,3,4]. In most cases, the injury occurs following an eccentric contraction of the quadriceps muscle, often during a fall or a sudden attempt to regain balance [2,3,4,5].

Diagnosis is predominantly clinical and is based on the presence of pain, an active knee extension deficit, and a palpable suprapatellar gap [2,6]. Magnetic resonance imaging (MRI) represents the diagnostic gold standard, allowing for an accurate assessment of the lesion and effective subsequent surgical planning [6].

The treatment of QTR is generally surgical, performed via transosseous repair or using suture anchors [2,6]. The literature reports overall satisfactory results following surgery [7,8]. However, several studies have highlighted the persistence of pain, stiffness, reduced sporting activity, and functional limitations in a significant proportion of patients, suggesting that perceived recovery does not always correlate with the clinical success of the tendon repair [7,8,9].

Despite the growing interest in Patient-Reported Outcome Measures (PROMs), evidence regarding quality of life and subjective knee function after quadriceps tendon repair remains limited [7,8,9,10]. Moreover, most available series include heterogeneous or predominantly younger cohorts, and specific evidence on patients over 50 years of age—who often present a distinct comorbidity profile and different functional demands—remains scarce. We therefore hypothesized that surgical repair of QTR in this population would yield satisfactory knee-specific functional recovery and a preserved quality of life at medium-term follow-up, despite the potential influence of age-related comorbidities. Therefore, the purpose of the present study is to evaluate the functional outcomes and quality of life of patients over 50 who underwent surgical repair for quadriceps tendon rupture using validated questionnaires (IKDC, Lysholm, and SF-36). The primary outcome was knee-specific functional recovery, assessed using the IKDC and Lysholm scores; secondary outcomes included health-related quality of life (SF-36), postoperative range of motion, residual extension lag, and complications, including tendon re-rupture.

## 2. Materials and Methods

A retrospective study was conducted to evaluate the clinical and functional outcomes, as well as the quality of life, of patients who underwent surgical repair for QTR. Patients treated for QTR at the authors’ institution between 1 January 2018, and 31 December 2024, with a minimum follow-up of 6 months (corresponding to the completion of the therapeutic-rehabilitation pathway), were included. This was a retrospective, single-center study: patients were identified through the hospital’s operative registry, and preoperative and perioperative variables were retrospectively extracted from clinical records. Clinical evaluation, range-of-motion assessment, and PROM questionnaires (administered via Google Forms) were collected at each patient’s final scheduled outpatient follow-up visit, at a minimum of 6 months after surgery. The study is reported in accordance with the STROBE (STrengthening the Reporting of OBservational studies in Epidemiology) statement for cohort studies; the completed STROBE checklist is provided as Supplementary Material (Table S1).

Exclusion criteria were the presence of associated knee injuries, associated patellar fractures, periprosthetic knee lesions, and revision surgeries for recurrent rupture.

Patients were identified through the hospital’s operative registry. Demographic data (age and sex), risk factors related to the type of injury (BMI, smoking habit, diabetes mellitus), and the time elapsed since the trauma (acute vs. chronic) were collected. At the final follow-up, data regarding the range of motion (ROM) of the operated knee and the potential use of walking aids were recorded. Additionally, data concerning postoperative complications (infections and re-ruptures) were collected. Active ROM of the operated knee was assessed at the final outpatient follow-up visit using a standard manual goniometer, with the patient positioned supine on the examination table. Measurements were performed by two knee-surgery-specialized orthopaedic surgeons (S.R. and M.C.). Active knee extension was recorded as 0° (full active extension) in all patients without a clinically detectable extension lag; the two patients with a measurable deficit were recorded as having an extension lag of 5° and 10°, respectively.

The postoperative rehabilitation protocol consisted of partial weight-bearing with crutches, permitted with the knee immobilized in a hinged brace locked in full extension for the first 30 postoperative days. During this same 30-day period, gradual unweighted (non-weight-bearing) mobilization exercises for the recovery of knee flexion were allowed, performed with the brace temporarily removed under supervision. From the third postoperative month, ambulation with an unlocked brace was permitted, followed by progressive weaning off the brace. Clinical follow-up included standardized evaluations at 30, 60, and 90 days after surgery.

Patient-Reported Outcome Measures (PROMs) were evaluated using three validated, self-administered questionnaires: the International Knee Documentation Committee Subjective Knee Form (IKDC), the Medical Outcomes Study 36-Item Short Form Health Survey (SF-36), and the Lysholm Knee Scoring Scale (Lysholm score).

The IKDC is a knee-specific questionnaire widely used to assess symptoms, joint function, and the ability to perform daily and sporting activities in patients with musculoskeletal knee pathologies [11,12]. The total score ranges from 0 to 100, with higher values indicating better perceived function [11].

Health-related quality of life was evaluated using the SF-36 questionnaire, one of the most widely used generic instruments for measuring perceived health status [13,14,15]. The questionnaire comprises 36 questions grouped into eight core domains, plus an additional single-item Health Transition domain that was also administered and analyzed in the present study; scores for each domain are expressed on a scale from 0 to 100, where higher scores indicate a better perceived quality of life [13]. Unlike the eight core domains, the Health Transition item does not contribute to the standard physical or mental component summary scores; it provides an independent, self-reported measure of the patient’s perceived change in overall health status relative to the previous year and was included to obtain an additional subjective indicator of the patient’s global psychophysical condition.

The Lysholm Knee Scoring Scale (Lysholm score) is a knee-specific questionnaire widely used for the subjective evaluation of knee symptoms and function, particularly following trauma and ligamentous pathologies. It includes domains such as limp, walking-aid use, locking, instability, pain, swelling, stair-climbing, and squatting [16]. The total score ranges from 0 to 100, with higher values indicating fewer symptoms and better function in activities of daily living.

Following informed consent to participate, the three questionnaires were administered digitally via Google Forms using the validated Italian versions [15,16,17]. Questionnaire scores were calculated according to the standard procedures provided by each assessment tool.

The combined use of the IKDC, SF-36, and Lysholm score allowed for the assessment of both knee-specific functional outcomes and the overall impact of the injury and surgical treatment on quality of life, thereby providing a multidimensional evaluation of patient-reported outcomes.

### Statistical Analysis

Continuous variables are expressed as mean ± standard deviation (SD) and range. Subgroup comparisons (patients younger than 65 years vs. those aged 65 years or older; transosseous tunnel vs. suture anchor technique) are reported for descriptive purposes only. Given the marked imbalance between the transosseous tunnel (*n* = 27) and suture anchor (*n* = 2) groups, no formal inferential statistical testing was performed to compare surgical techniques, and these figures should not be interpreted as evidence of equivalence or superiority between the two methods. To evaluate the potential for selection bias arising from the high rate of questionnaire non-completion, baseline characteristics (age, BMI, diabetes mellitus, and smoking habit) of patients included in the final analysis were compared with those of patients not included, using Welch’s *t*-test for continuous variables and Fisher’s exact test for categorical variables. All statistical analyses were performed using Jamovi (v2.6.26, The jamovi project, 2025). The normality of continuous variables was assessed using the Shapiro–Wilk test. Age, BMI, IKDC, and the SF-36 Physical Functioning, Vitality, and General Health domains were normally distributed (*p* > 0.05), whereas the Lysholm score, postoperative flexion ROM, and the remaining SF-36 domains (Role Physical, Role Emotional, Mental Health, Social Functioning, Bodily Pain, and Health Transition) significantly deviated from a normal distribution (*p* < 0.05). For consistency and comparability with the existing orthopaedic literature on this topic, all continuous variables are reported as mean ± SD; for variables that deviated from normality, the median and range are additionally reported in Table 1 and Table 2, and results for these variables should be interpreted with this in mind. Patients with incomplete data for any of the required questionnaires or clinical parameters were excluded from the final analysis; no imputation was performed for missing data. The 65-year age cut-off used for subgroup stratification was defined a priori, in order to explore whether age at the time of injury was associated with differences in functional outcomes within the over-50 cohort studied; given the limited subgroup sample sizes, these comparisons are descriptive and hypothesis-generating rather than confirmatory. To explore the relationship between knee-specific functional outcomes and health-related quality of life, Spearman’s rank correlation coefficients, with 95% confidence intervals derived via Fisher’s z-transformation, were calculated between the IKDC and Lysholm scores and each SF-36 domain. Since this was a retrospective study, a sensitivity power analysis was additionally conducted to contextualize the statistical power of the analyses, indicating that, with the achieved sample size and a two-sided α = 0.05, the study had 80% power to detect between-group differences corresponding to a Cohen’s d of at least 0.71 (included vs. non-included comparison) and 1.10 (age-subgroup comparison), and Spearman correlation coefficients of at least approximately 0.50; smaller effects could not be reliably excluded, and non-significant findings should be interpreted accordingly.

## 3. Results

Between January 2018 and December 2024, 65 patients with QTR underwent surgical treatment at the CTO Hospital in Turin. Of these 65 initially selected patients, 4 were excluded due to recurrent ruptures from a previous injury. Of the remaining 61 patients, 29 (47.5%) fully completed all questionnaires required by the protocol and were included in the final analysis, while the remaining 32 (52.5%) were excluded because of incomplete questionnaire data; combined with the 4 patients excluded for recurrent rupture, a total of 36 of the original 65 patients (55.4%) were not included in the final analysis. To assess the potential for selection bias, the baseline characteristics of patients included in the final analysis were compared with those of all patients not included (Table 1). Patients not included in the analysis were significantly older than those included (67.5 ± 9.9 vs. 62.7 ± 7.6 years; *p* = 0.032), while no significant differences were observed for body mass index (27.5 ± 3.0 vs. 28.6 ± 4.0 kg/m^2^; *p* = 0.224), diabetes mellitus (12.5% vs. 6.9%; *p* = 0.674), or smoking habit (14.8% vs. 7.7%; *p* = 0.669). This finding suggests that older patients may have been relatively under-represented in the final analysis, a point further addressed in the Limitations section (Figure 1).

The study cohort comprised 27 males and 2 females. At the time of surgery, the mean age of the evaluated patients was 62.74 ± 7.61 years (range, 50–80). Among the 29 examined patients, 1 patient presented with a bilateral QTR. This patient underwent bilateral repair and contributed two separate ROM measurements, one per operated knee; questionnaire-based outcomes (IKDC, Lysholm, SF-36), by contrast, were recorded once per patient, reflecting the patient’s overall subjective perception rather than knee-specific scores. Consequently, the ROM analysis is based on 30 operated knees, while all PROM-based analyses are based on 29 patients.

Regarding risk factor analysis, 2 patients were affected by diabetes mellitus and 2 had a smoking habit. At the time of surgery, the mean recorded BMI was 28.6 ± 3.99 kg/m^2^ (range, 19.15–38.42); specifically, 86.7% of the patients had a BMI greater than 25. The mean time elapsed from injury to surgery was 13.1 ± 7.41 days (range, 1–32 days).

Of the 29 patients examined, 27 were treated with a transosseous tunnel suture using FiberWire^®^ No. 5, Arthrex, Naples, FL, USA (Figure 2), while 2 were treated using suture anchors; given this marked imbalance, the two techniques are described separately for descriptive purposes and were not statistically compared (see Statistical Analysis). All patients were evaluated at a minimum of 6 months postoperatively, with a final mean follow-up of 9.17 months (range, 6–11 months).

The mean postoperative flexion range of motion (ROM) was 102.4° ± 14.98° (range, 60–120°). At the end of the rehabilitation protocol, 2 patients presented a residual extension lag of 5° and 10°, respectively. Active knee extension was complete (0°) in the remaining 27 patients (93.1%). Only one patient (1/29) experienced a surgical wound infection, which resolved with antibiotic therapy. At the final follow-up, all patients were ambulating without the use of walking aids (Table 3, Figure 3).

The mean score obtained from the IKDC evaluation questionnaire was 63.2 ± 15.83 (range, 27.6–93.1). The mean Lysholm score was 84.3 ± 9.35 (range, 60–96) (Figure 4). The clinical score results were also stratified according to the a priori-defined age cut-off of 65 years (see Statistical Analysis). Patients younger than 65 years (*n* = 17) achieved a mean IKDC score of 64.1 and a mean Lysholm score of 86.4; patients aged 65 years or older (*n* = 12) showed a mean IKDC score of 62.1 and a mean Lysholm score of 81.3 (Figure 4 and Figure 5). Age-stratified results for the SF-36 domains, including the Health Transition domain (mean 63.2 in patients < 65 years vs. 52.1 in patients ≥ 65 years), are reported in Table 3.

Administration of the SF-36 questionnaire yielded the results shown in Table 2.

To further explore the relationship between functional outcomes and health-related quality of life, Spearman’s rank correlation coefficients were calculated between the IKDC and Lysholm scores and each SF-36 domain. Both questionnaires showed their strongest, statistically significant correlations with Physical Functioning (ρ = 0.63 and 0.82, respectively) and Bodily Pain (ρ = 0.78 and 0.64); moderate, significant correlations were also observed with Vitality and General Health for both instruments, with Social Functioning for the IKDC, and with Role Physical for the Lysholm score. Correlations with Role Emotional, Mental Health, and Health Transition were weaker and did not reach statistical significance, with 95% confidence intervals spanning zero; given the achieved sample size (see Statistical Analysis), these findings should be regarded as inconclusive rather than as evidence of an absent relationship. No significant correlation was observed between patient age and either the IKDC or Lysholm scores.

## 4. Discussion

The primary objective of our study was to evaluate the clinical and functional outcomes of quadriceps tendon repair, with a specific focus on the impact that the injury and subsequent surgery can have on the patient’s quality of life. This work demonstrates that quadriceps tendon repair using the transosseous tunnel suture technique yields good outcomes in an over-50 population (range, 50–80 years). At a mean follow-up of 9.17 months, we observed an IKDC score of 63.2 points and a Lysholm score of 83.15 points, associated with an absence of re-ruptures and a low incidence of residual extension lag.

QTR predominantly affects male patients in their fifth and sixth decades of life and is frequently associated with tendon degenerative phenomena and systemic comorbidities [18]. In the systematic review by Ciriello et al., which included 319 patients with a mean age of 57 years, the most frequent traumatic mechanism was a simple fall, and the poorest outcomes were associated with diagnostic and therapeutic delays. Furthermore, the authors highlighted that residual quadriceps atrophy and strength deficits are common despite overall good clinical results [10].

It is well-established that obesity, diabetes, chronic renal failure, and other metabolic conditions can promote tendon degeneration and are responsible for an increased risk of quadriceps tendon rupture [3,4,19]. A particularly interesting element is represented by the characteristics of the population analyzed in this study. Eighty-six-point seven percent (86.7%) of the patients were overweight or obese, with a mean BMI of 28.3 kg/m^2^, whereas diabetes mellitus was present in only two cases. Despite these predisposing factors, our functional outcomes were good, suggesting that a stable surgical repair can at least partially offset the potential negative effect of systemic comorbidities on the healing process. Langenhan et al. [20] conducted a retrospective multicenter study analyzing 191 patients with QTR, a mean BMI of 30.4, and a mean age of 62.3 years; this patient cohort, similar to ours in terms of obesity prevalence and mean age, showed favorable clinical outcomes, documented by IKDC and Lysholm scores of 67.4 and 74.8, respectively. These results align with those reported by other studies conducted in populations characterized by a high BMI [21,22].

Regarding the recovery of range of motion in the operated knee, the final mean flexion was 98.5°, which is lower than the values reported in other recent studies [21,23,24]. Concerning knee extension, only two patients presented a residual, clinically detectable extension lag at the final follow-up, while active extension was complete in the remaining patients; however, this reflects the clinical range-of-motion assessment only, since quadriceps strength was not measured instrumentally in this study. This result appears favorable compared to available evidence reporting variable percentages of residual extensor lag and persistent strength deficits even in the presence of good clinical outcomes. Indeed, Konrath et al. and Ciriello et al. [10,25] emphasized that the complete recovery of quadriceps strength represents one of the most challenging aspects of treating these injuries. Despite the not fully physiological flexion ROM value, the patients achieved satisfactory IKDC and Lysholm scores; in the absence of an instrumented strength assessment, however, this observation should be regarded as hypothesis-generating rather than evidence that clinical extensor recovery outweighs the importance of full joint mobility.

The results of the present study are broadly consistent with the main analyses available in the literature, although the following comparisons are based on point estimates reported by each study rather than on formal statistical testing, since patient-level data were not available. Konrath et al. [25] reported a Lysholm score of 86 points at a four-year follow-up in patients with a mean age of 56 years. Similarly, Wenzl et al. [26] reported a mean Lysholm score of 92.5 points, while Boudissa et al. [27] described mean values of 93.7 points. The value of 83.15 points obtained in our case series thus appears comparable to, or moderately lower than, the values reported in these previous series [28,29,30]. Analyzing the results of the subjective IKDC questionnaire, the present study reveals that the mean score of 63.2 was lower than that reported in several literature studies [9,20,28,30,31].

An additional strength of the present study is the evaluation of quality of life using the SF-36 questionnaire. Although this instrument is widely used in orthopedics, its application in case series dedicated to QTRs is relatively limited. Our patients showed high scores in the domains of physical functioning (84.1), role limitations due to physical problems (80.3), role limitations due to emotional problems (90.8), and bodily pain (79.0), indicating a substantial recovery of daily function and social participation. The comparison between functional domains and those relating to the general perception of health is particularly interesting. Although patients achieved excellent functional outcomes, the scores for general health (67.9) and perceived health transition (58.6) were lower compared to the other domains. This discrepancy suggests that global health perception is likely influenced by age and associated comorbidities rather than the outcomes of the tendon repair itself.

In recent years, scientific interest has focused on comparing the techniques used to treat this type of injury. Although biomechanical studies have demonstrated greater initial construct strength with anchor repairs, available clinical results have not shown a clear superiority of one technique over the other [28]. In the comparative study by Plesser et al. [30], for instance, no significant differences were observed in IKDC scores, Lysholm scores, pain, or complication rates between transosseous and anchor repairs. Similarly, the systematic review by Ciriello et al. [10] concluded that the type of surgical repair does not significantly influence final clinical outcomes. Within our analysis, it was not possible to perform an effective comparison between the two types of repair techniques because the suture anchor technique was performed in only two cases, unlike the transosseous tunnel suture technique. The two patients treated with suture anchors showed excellent clinical and functional results; however, it is important to emphasize that these were two young (57 and 58 years old), active individuals with no risk factors; given the extremely small sample size (*n* = 2), this observation should be regarded as hypothesis-generating only and does not support a causal relationship between patient characteristics and outcome.

In this context, the results obtained with the transosseous tunnel suture technique demonstrated that the traditional technique could ensure good clinical and functional results. The absence of re-ruptures observed in our series appears particularly favorable when compared to the rates reported in the literature, which generally range between 2% and 6% [10].

In addition to the complexity of comparing the two techniques used, the primary limitations of this study reside in its retrospective design and the absence of a control group, which precludes a direct comparison with alternative repair methods. Furthermore, the small sample size, while reflecting the rarity of the pathology, reduces the statistical power of the analyses. A sensitivity power analysis indicated that, with the achieved sample size, the study had 80% power to detect only between-group differences of at least a large effect size (Cohen’s d ≥ 0.71–1.10, depending on the comparison) and correlations of |ρ| ≥ 0.50; smaller effects could not be reliably excluded, and non-significant findings throughout this study should be interpreted with this limitation in mind. The mean follow-up of 9.17 months, although sufficient to capture the completion of the structured rehabilitation pathway, is shorter than the 12- to 24-month benchmark commonly adopted in the literature to assess definitive functional outcomes after quadriceps tendon repair; therefore, the functional and quality-of-life results reported here should be regarded as early-to-medium-term findings, and longer follow-up would be needed to confirm their durability over time. A particularly relevant limitation is that more than half of the initially screened cohort (36 of 65 patients, 55.4%) did not complete the required questionnaires or were otherwise not included in the final analysis, corresponding to 32 of the 61 eligible patients (52.5%). A comparison of baseline characteristics between included and non-included patients (Table 1) showed that non-included patients were significantly older (67.5 ± 9.9 vs. 62.7 ± 7.6 years; *p* = 0.032), while BMI, diabetes mellitus, and smoking habit did not differ significantly between groups. This finding indicates that a selection bias related to age cannot be excluded, and that the reported functional and quality-of-life outcomes may be more representative of relatively younger patients within the over-50 population studied; this should be taken into account when interpreting the results. Additionally, PROMs were self-administered through a non-standardized, unmasked digital platform (Google Forms), without independent verification of clinical measures such as range of motion; this data-collection approach may have introduced further bias and should be regarded as a further methodological limitation. No a priori sample size calculation was performed, given the retrospective design and the rarity of the pathology; this represents an additional limitation of the study. It should also be noted that preoperative PROM scores were not available; therefore, the reported IKDC, Lysholm, and SF-36 results reflect the patients’ postoperative status rather than the magnitude of improvement from baseline, and should be interpreted accordingly. Finally, the lack of an instrumental assessment of muscle strength prevents a direct correlation between clinical outcomes and the biomechanical recovery of the extensor mechanism.

## 5. Conclusions

In this retrospective, single-center, uncontrolled case series, patients over 50 years of age who underwent quadriceps tendon repair and completed a medium-term follow-up (mean 9.17 months) showed overall satisfactory clinical and functional outcomes, with good knee mobility, autonomy in activities of daily living, and a substantially preserved quality of life. These findings suggest that surgical repair of the quadriceps tendon may contribute to restoring joint function and supporting psychophysical well-being in this age group, although this uncontrolled case series cannot establish a causal or comparative claim of treatment efficacy. Given the retrospective and uncontrolled design, the small and markedly imbalanced surgical-technique subgroups, and the exclusion of more than half of the originally eligible cohort because of incomplete questionnaire data, these results should be interpreted with caution and should not be regarded as definitive evidence of the reliability, efficacy, or superiority of this procedure over alternative techniques. Larger, prospective, controlled studies with systematic follow-up are needed to confirm these preliminary findings.

## Figures and Tables

**Figure 1:**
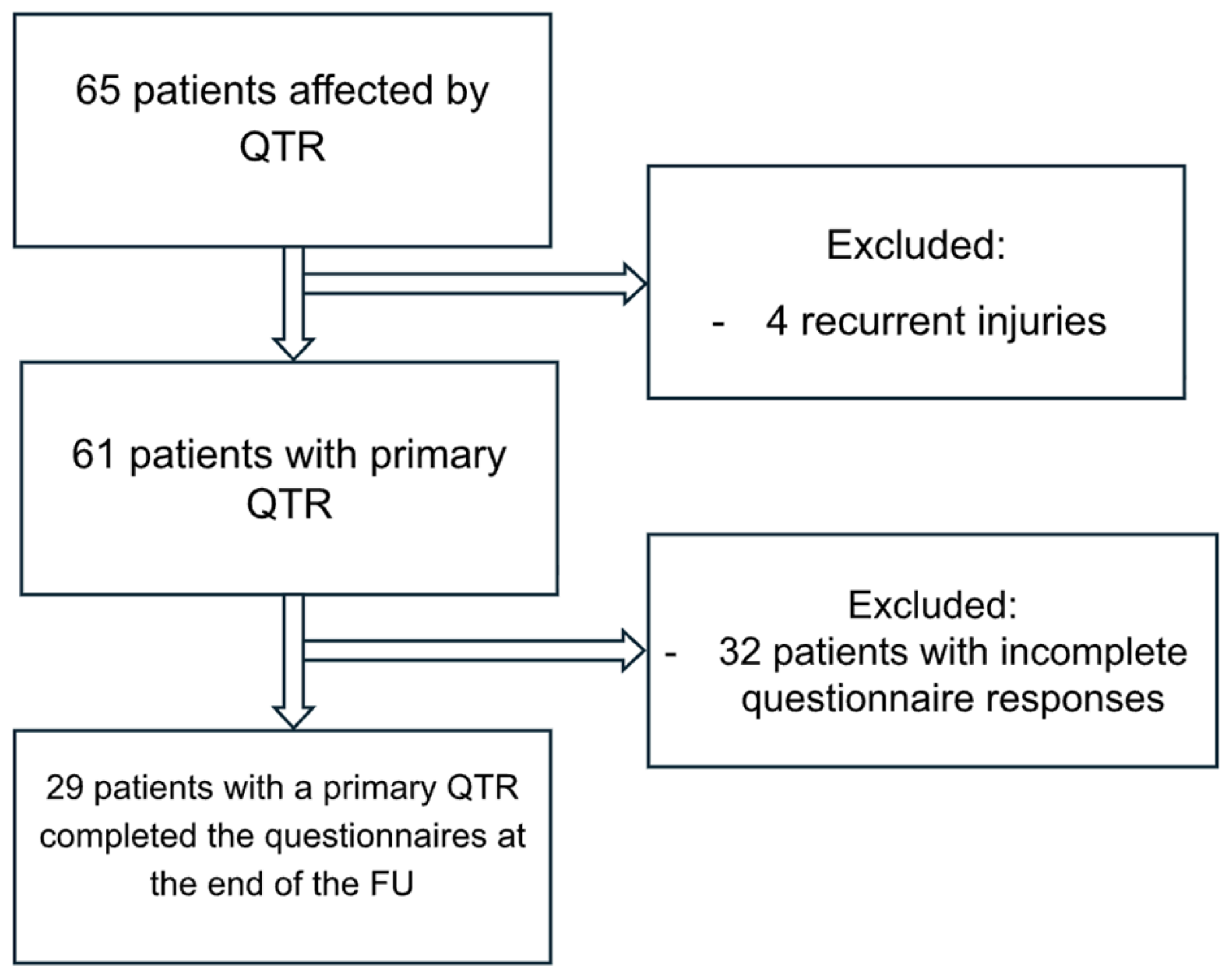
QTR: quadriceps tendon rupture; FU: follow-up.

**Figure 2:**
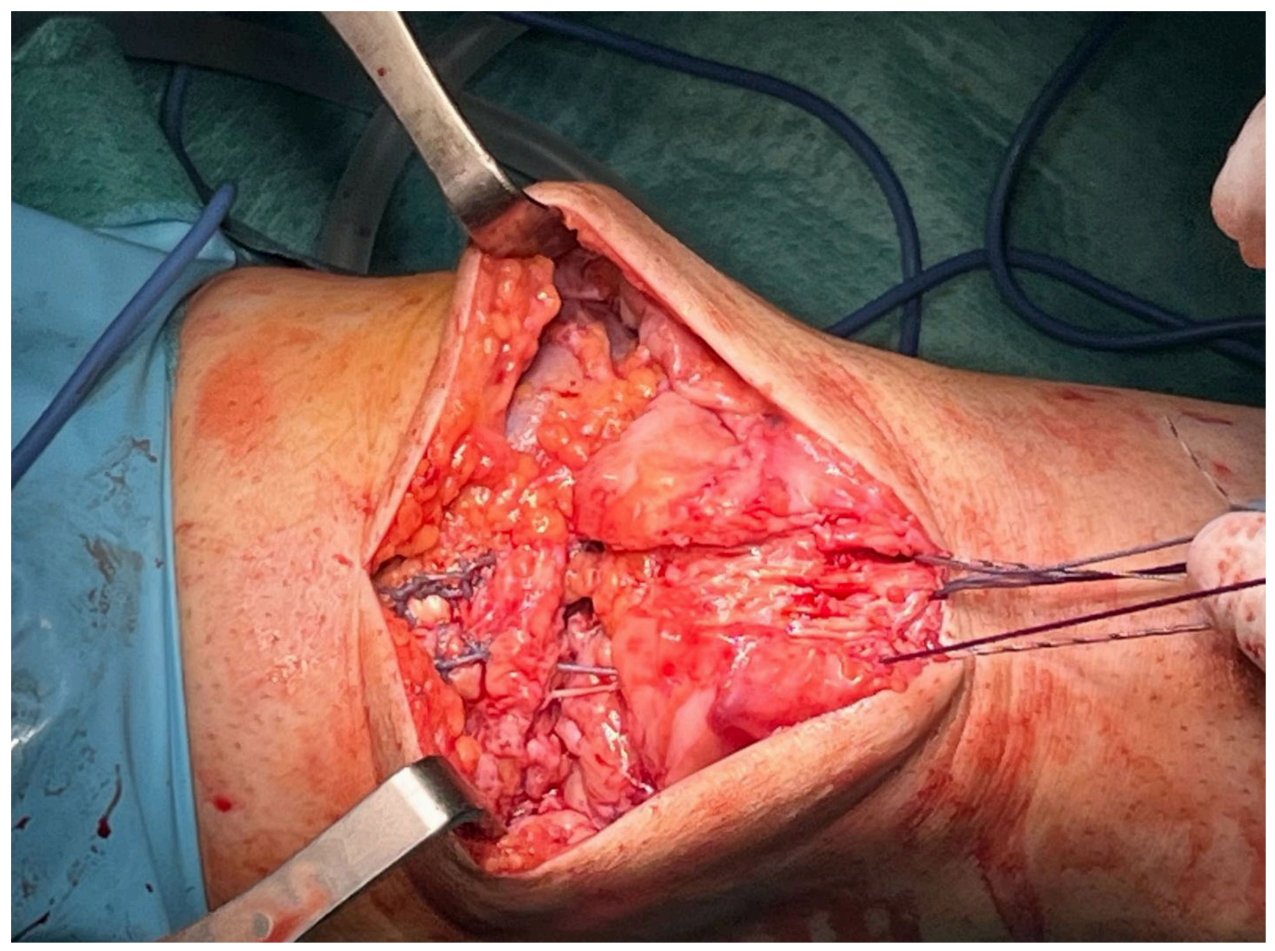
Quadriceps Tendon Repair Using a Transosseous Suture Technique.

**Figure 3:**
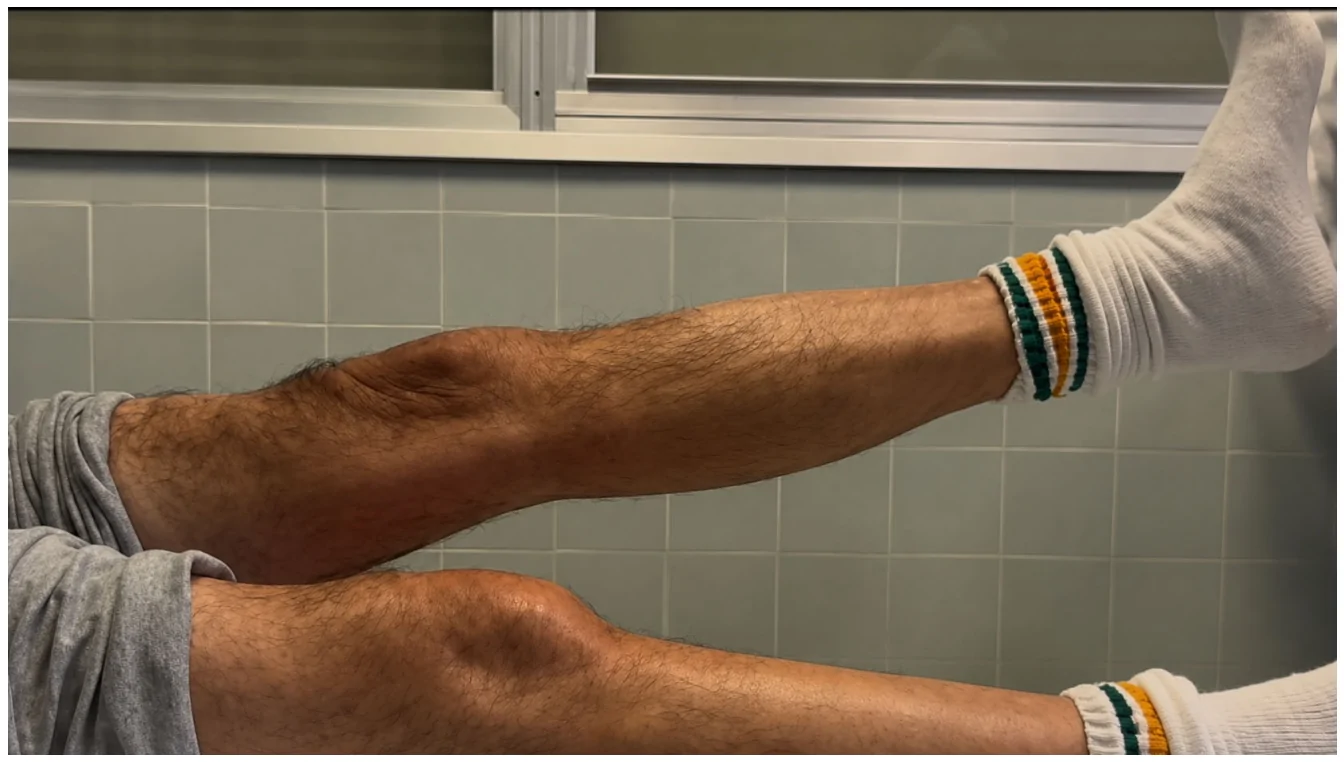
Clinical image at final follow-up, showing full active extension of the knee in a patient who underwent quadriceps tendon repair.

**Figure 4:**
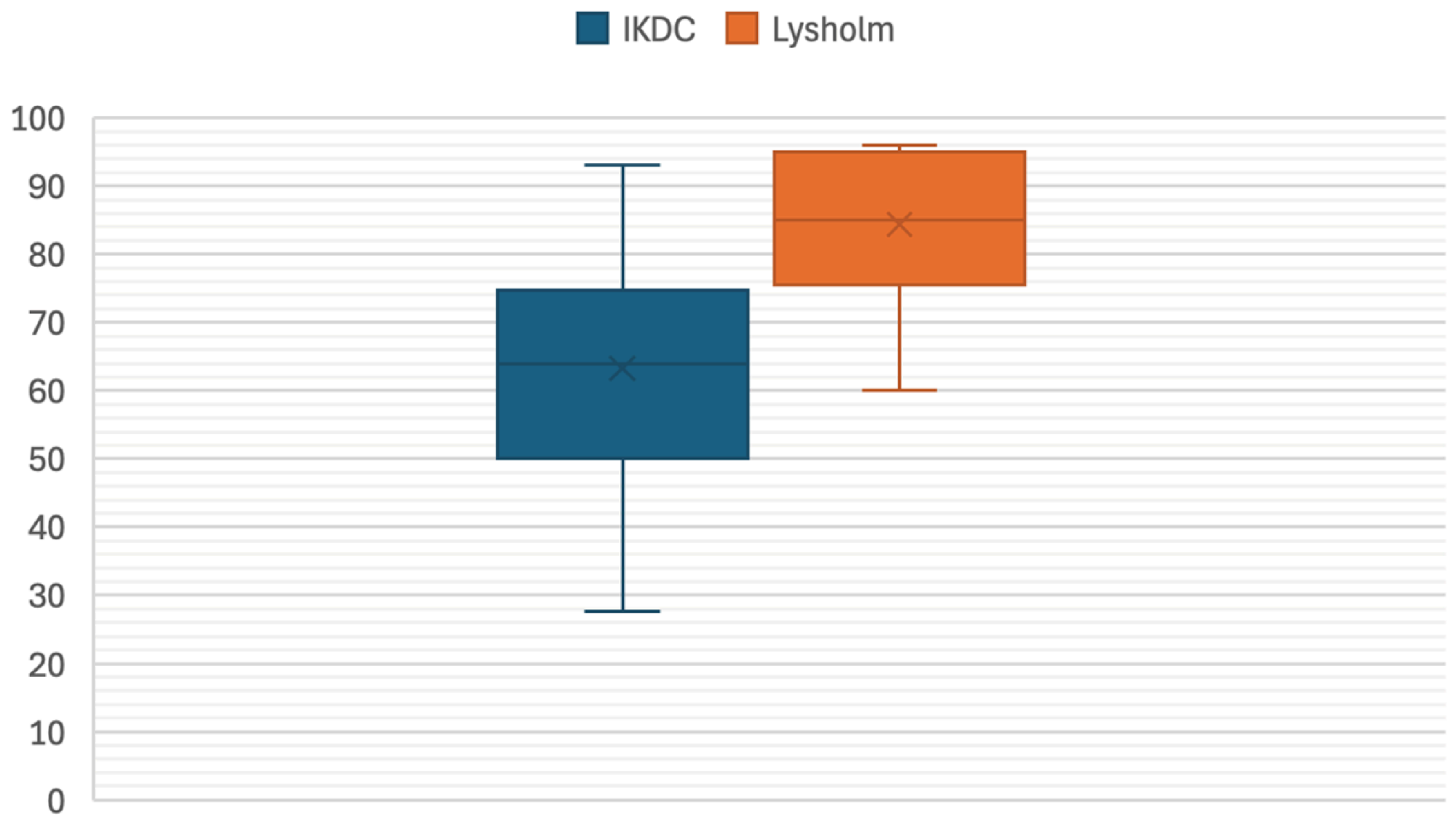
Box plots showing the distribution of IKDC and Lysholm scores in the study population. The central line represents the median, the box indicates the interquartile range (IQR), whiskers represent the minimum and maximum values.

**Figure 5:**
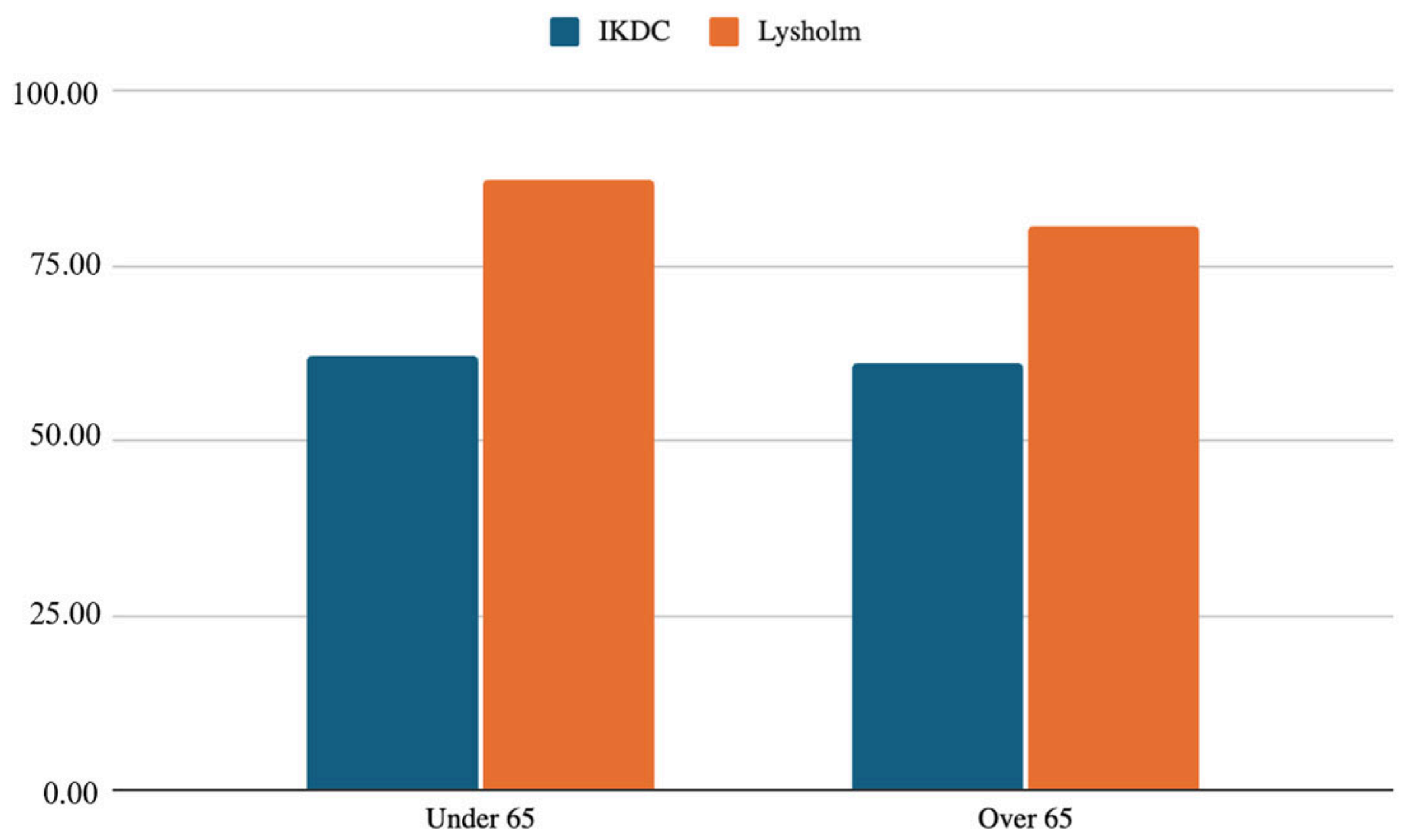
Comparison of the mean IKDC and Lysholm questionnaire scores between patients aged < 65 years and ≥65 years. Values represent group means; given the limited subgroup sample sizes, no measures of variability or inferential statistical comparisons are presented, and these results should be considered descriptive and hypothesis-generating only.

**Table 1:** Comparison of baseline characteristics between patients included in the final analysis and patients not included (due to recurrent rupture from a previous injury or incomplete questionnaire data). Continuous variables were compared using Welch’s *t*-test; categorical variables were compared using Fisher’s exact test. BMI, body mass index; SD, standard deviation.

Variable	Included in Analysis (*n* = 29)	Not Included (*n* = 36)	*p*-Value
Age (years, mean ± SD)	62.7 ± 7.6	67.5 ± 9.9	0.032
BMI (kg/m^2^, mean ± SD)	28.6 ± 4.0	27.5 ± 3.0	0.224
Diabetes mellitus, *n* (%)	2 (6.9%)	4 (12.5%)	0.674
Smoking habit, *n* (%)	2 (7.7%)	4 (14.8%)	0.669

**Table 2:** Mean scores, standard deviation, and range obtained by patients in the different domains of the SF-36 questionnaire at final follow-up. Scores for each domain are expressed on a scale from 0 to 100, where higher values indicate a better health-related quality of life.

	Mean	STD DEV	Median; Range	Mean Under 65	Mean over 65
Physical Functioning	84.1	12.18	Median 85.0; range 55–100	85.9	81.7
Role Physical	80.3	34.17	Median 100.0; range 0–100	94.4	60.4
Role Emotional	90.8	25.05	Median 100.0; range 0–100	94.2	86.1
Vitality	66.0	16.44	Median 60.0; range 35–100	65.3	67.1
Mental Health	74.1	17.62	Median 76.0; range 12–100	73.6	74.7
Social Functioning	78.2	17.90	Median 87.5; range 25–100	80.3	75.2
Bodily Pain	79.0	22.64	Median 90.0; range 23–100	81.0	76.2
General Health	67.9	18.54	Median 70.0; range 20–100	72.4	61.7
Health Transition	58.6	25.25	Median 50.0; range 25–100	63.2	52.1

**Table 3:** Demographic data, clinical parameters, and complications of the study population at final follow-up (*n* = 29). Abbreviations: BMI, body mass index; IKDC, International Knee Documentation Committee; ROM, range of motion.

N° of Patients	29
Age	62.74 ± 7.61; median 63.9 (range, 50–80)
BMI	28.6 ± 3.99; median 28.1 (range, 19.15–38.42)
Technique	Transosseous tunnel 27—Suture anchor 2
IKDC	63.2 ± 15.83; median 64.0 (range, 27.6–93.1)
Lysholm	84.3 ± 9.35; median 85.0 (range, 60–96)
ROM (flexion)	102.4 ± 14.98; median 100.0 (range, 60–120)
Extension lag	2 (6.9%)
Infection	1 (3.4%)

## Data Availability

The original contributions presented in this study are included in the article/Supplementary Material. Further inquiries can be directed to the corresponding author.

## Supplementary Materials

The following supporting information can be downloaded at: https://www.mdpi.com/article/10.3390/jfmk11040381/s1, Table S1: STROBE Statement—checklist of items that should be included in reports of cohort studies [32].

## Abbreviations

The following abbreviations are used in this manuscript:

QTR	Quadriceps Tendon Rupture
IKDC	International Knee Documentation Committee Subjective Knee Form
BMI	Body Mass Index
SF-36	Medical Outcomes Study 36-Item Short Form Health Survey
ROM	Range of motion
PROMs	Patient-Reported Outcome Measures
