# Supplementary material for: Functional Outcomes and Quality of Life in Patients over 50 Years Old Treated with Quadriceps Tendon Repair: A Retrospective Study
Source: J Funct Morphol Kinesiol. 2026 Sep 24;11(4):381. doi: 10.3390/jfmk11040381 (PMC13692438; doi:10.3390/jfmk11040381)
Supplement: Supplementary file 1 [file jfmk-11-00381-s001.zip › jfmk-4473589-supplementary.pdf]

Table S1. STROBE Statement

Checklist of items that should be included in reports of cohort studies

Manuscript: "Functional outcomes and quality of life in patients over 50 years old treated with quadriceps tendon repair: a retrospective study" – submitted to Journal of Functional Morphology and Kinesiology.

Item No.	Recommendation	Reported in manuscript (section / paragraph)
Title and abstract		
1a	Indicate the study's design with a commonly used term in the title or the abstract.	Title ("...a retrospective study"); Abstract, Materials and Methods.
1b	Provide in the abstract an informative and balanced summary of what was done and what was found.	Abstract.
Introduction		
2	Explain the scientific background and rationale for the investigation being reported.	Introduction, paragraphs 1–2.
3	State specific objectives, including any prespecified hypotheses.	Introduction, final paragraph (aim of the study; primary/secondary outcomes).
Methods		
4	Present key elements of study design early in the paper.	Methods, opening paragraph: retrospective, single-center study; PROM questionnaires administered via Google Forms at each patient's final scheduled outpatient follow-up visit; STROBE adherence stated.
5	Describe the setting, locations, and relevant dates, including periods of recruitment, exposure, follow-up, and data collection.	Methods, opening paragraph (single center – authors' institution; January 2018–December 2024; minimum 6-month follow-up).
6a	Give the eligibility criteria, and the sources and methods of selection of participants.	Methods: inclusion criteria and exclusion criteria (associated knee injuries, patellar fractures, periprosthetic lesions, revision surgery); patients identified through the hospital's operative registry.

Item No.	Recommendation	Reported in manuscript (section / paragraph)
6b	For matched studies, give matching criteria and number of exposed and unexposed.	Not applicable – this is not a matched study.
7	Clearly define all outcomes, exposures, predictors, potential confounders, and effect modifiers. Give diagnostic criteria, if applicable.	Methods: ROM, complications, PROMs (IKDC, Lysholm, SF-36) defined; Statistical Analysis: 65-year age cut-off defined a priori.
8	For each variable of interest, give sources of data and details of methods of assessment. Describe comparability of assessment methods if more than one group.	Methods: ROM assessed with standard goniometer by two knee-surgery-specialized orthopaedic surgeons; PROMs administered digitally via Google Forms using validated Italian versions [refs].
9	Describe any efforts to address potential sources of bias.	Statistical Analysis and Discussion/Limitations: comparison of baseline characteristics between included and non-included patients (Table 2) to assess selection bias.
10	Explain how the study size was arrived at.	Methods/Results: all consecutive patients treated at the institution during the study period who met eligibility criteria were included; no a priori sample-size calculation was performed (noted as a limitation).
11	Explain how quantitative variables were handled in the analyses. If applicable, describe which groupings were chosen and why.	Statistical Analysis: continuous variables reported as mean ± SD (median and range additionally reported for non-normally distributed variables, Tables 1 and 3); 65-year age cut-off for subgroup analysis defined a priori and rationale explained.
12a	Describe all statistical methods, including those used to control for confounding.	Statistical Analysis: normality of continuous variables assessed with the Shapiro-Wilk test; Welch's t-test and Fisher's exact test for comparison of included vs. non-included patients; no confounder adjustment performed (descriptive case series).

Item No.	Recommendation	Reported in manuscript (section / paragraph)
12b	Describe any methods used to examine subgroups and interactions.	Statistical Analysis: age (<65 vs. ≥65 years) and surgical-technique subgroups analyzed descriptively; not formally tested for interaction.
12c	Explain how missing data were addressed.	Statistical Analysis: patients with incomplete data for any required questionnaire or clinical parameter were excluded from the final analysis; no imputation was performed.
12d	If applicable, explain how loss to follow-up was addressed.	Statistical Analysis / Limitations: baseline comparison of completers vs. non-completers (Table 2); discussed as a limitation given the high non-completion rate.
12e	Describe any sensitivity analyses.	Not performed; noted as a direction for future research.
Results		
13a	Report the numbers of individuals at each stage of the study (e.g., eligible, included, completing follow-up, analysed).	Results, opening paragraph: 61 eligible patients → 29 (47.5%) completed all required questionnaires and were analyzed.
13b	Give reasons for non-participation at each stage.	Discussion/Limitations: non-completion of follow-up questionnaires; specific individual reasons (e.g., unreachable, refusal, death) were not systematically recorded, noted as a limitation.
13c	Consider use of a flow diagram.	Not currently included; can be added at the Editor's request.
14a	Give characteristics of study participants and information on exposures and potential confounders.	Table 1; Results, demographic and risk-factor paragraph.
14b	Indicate the number of participants with missing data for each variable of interest.	No missing data: all variables, including the Lysholm score, were complete for the full cohort (N = 29).

Item No.	Recommendation	Reported in manuscript (section / paragraph)
14c	Summarise follow-up time.	Results: mean follow-up 9.17 months (range, 6–11 months).
15	Report numbers of outcome events or summary measures.	Results, Table 1, Table 3.
16a	Give unadjusted estimates and, if applicable, confounder-adjusted estimates and their precision.	Results/Table 1/Table 3: mean ± SD reported for all outcomes, with median and range additionally reported for non-normally distributed variables; no adjusted estimates (descriptive case series).
16b	Report category boundaries when continuous variables were categorized.	Statistical Analysis / Results: 65-year age cut-off explicitly reported.
16c	If relevant, translate estimates of relative risk into absolute risk.	Not applicable – no relative-risk estimates were calculated in this descriptive case series.
17	Report other analyses done (e.g., subgroup, sensitivity analyses).	Results: age-stratified IKDC, Lysholm, and SF-36 domain scores (Figure 4–5, Table 3).
Discussion		
18	Summarise key results with reference to study objectives.	Discussion, opening paragraph.
19	Discuss limitations of the study, taking into account sources of potential bias or imprecision. Discuss direction and magnitude of any potential bias.	Discussion, Limitations paragraph: retrospective/uncontrolled design, selection bias from non-completion (Table 2), non-standardized digital PROM collection, absence of baseline PROM data, no instrumented strength testing.
20	Give a cautious overall interpretation considering objectives, limitations, multiplicity of analyses, results from similar studies, and other relevant evidence.	Discussion, throughout; Conclusions.
21	Discuss the generalisability (external validity) of the study results.	Conclusions: results not to be regarded as definitive evidence given retrospective, single-center, uncontrolled design and imbalanced subgroups.

Item No.	Recommendation	Reported in manuscript (section / paragraph)
Other information		
22	Give the source of funding and the role of funders for the present study.	Funding statement: “This research received no external funding.”

Note: this checklist is based on the STROBE Statement for cohort studies (von Elm E, Altman DG, Egger M, Pocock SJ, Gøtzsche PC, Vandenbroucke JP; STROBE Initiative. Ann Intern Med. 2007;147(8):573-577), adapted for this manuscript.
